# Iodine Intake from Food and Iodized Salt as Related to Dietary Salt Consumption in the Italian Adult General Population

**DOI:** 10.3390/nu13103486

**Published:** 2021-09-30

**Authors:** Roberto Iacone, Paola Iaccarino Idelson, Ornella Russo, Chiara Donfrancesco, Vittorio Krogh, Sabina Sieri, Paolo Emidio Macchia, Pietro Formisano, Cinzia Lo Noce, Luigi Palmieri, Daniela Galeone, Domenico Rendina, Ferruccio Galletti, Andrea Di Lenarda, Simona Giampaoli, Pasquale Strazzullo, on behalf of the MINISAL-GIRCSI Study Group

**Affiliations:** 1 Department of Clinical Medicine and Surgery, Federico II University of Naples Medical School, 80131 Naples, Italy; ornella.russo@unina.it (O.R.); pmacchia@unina.it (P.E.M.); domenico.rendina@unina.it (D.R.); galletti@unina.it (F.G.); strazzul@unina.it (P.S.); 2 Department of Cardiovascular, Endocrine-Metabolic Diseases and Aging, Istituto Superiore di Sanità, 00161 Rome, Italy; chiara.donfrancesco@iss.it (C.D.); cinzia.lonoce@iss.it (C.L.N.); luigi.palmieri@iss.it (L.P.); 3 Epidemiology and Prevention Unit, Fondazione IRCCS Istituto Nazionale dei Tumori di Milano, 20133 Milano, Italy; vittorio.krogh@istitutotumori.mi.it (V.K.); sabina.sieri@istitutotumori.mi.it (S.S.); 4 Department of Translational Medical Science, Federico II University of Naples Medical School, 80131 Naples, Italy; pietro.formisano@unina.it; 5 Italian Ministry of Health, Center for Disease Prevention and Control, 00161 Rome, Italy; d.galeone@sanita.it; 6 ANMCO, Italian Association of Hospital Cardiology, 50121 Florence, Italy; dilenar@units.it; 7 Cardiovascular Center, University Hospital and Health Services, 34122 Trieste, Italy; 8 Former Department of Cardiovascular, Endocrine-Metabolic Diseases and Aging, Istituto Superiore di Sanità, 00161 Rome, Italy; simona.giampaoli@iss.it

**Keywords:** iodine prophylaxis, iodine deficiency disorders, thyroid, adult age, iodine intake, 24 h urinary excretion, salt intake, salt restriction, iodized salt

## Abstract

Since the Italian iodoprophylaxis strategy is based on the use of iodized salt, we assessed the relationship between dietary salt consumption and iodine intake in the Italian adult population. We estimated the relative contribution given by the use of iodized salt and by the iodine introduced by foods to the total iodine intake. The study population included 2219 adults aged 25–79 years (1138 men and 1081 women) from all Italian regions, participating to the Osservatorio Epidemiologico Cardiovascolare/Health Examination Survey 2008–2012 (OEC/HES), and examined for sodium and iodine intake in the framework of the MINISAL-GIRCSI Programme. Dietary sodium and total iodine intake were assessed by the measurement of 24 h urinary excretion, while the EPIC questionnaire was used to evaluate the iodine intake from food. Sodium and iodine intake were significantly and directly associated, upon accounting for age, sex, and BMI (Spearman rho = 0.298; *p* < 0.001). The iodine intake increased gradually across quintiles of salt consumption in both men and women (*p* < 0.001). The European Food Safety Authority (EFSA) adequacy level for iodine intake was met by men, but not women, only in the highest quintile of salt consumption. We estimated that approximately 57% of the iodine intake is derived from food and 43% from salt. Iodized salt contributed 24% of the total salt intake, including both discretionary and non-discretionary salt consumption. In conclusion, in this random sample of the Italian general adult population examined in 2008–2012, the total iodine intake secured by iodized salt and the iodine provision by food was insufficient to meet the EFSA adequate iodine intake.

## 1. Introduction

The amount of iodine provided by food has proved insufficient to fulfil the physiological needs in many regions of the world, including Italy: thus, for the prevention of iodine insufficiency and related thyroid disorders the World Health Organization (WHO) recommends fortification of food-grade salt with iodine [1,2]. Salt is considered an appropriate vehicle for fortification with iodine because of its widespread consumption, low cost, and lack of effect of the added iodine on food palatability [3].

In Italy, the legislation on iodoprophylaxis delivered in 2005 established the addition of potassium iodate (30 mg/kg) to food-grade salt and the mandatory availability of iodised salt in food shops, supermarkets, and public catering [4]. Since 2009 the National Observatory for the Monitoring of Iodoprophylaxis (OSNAMI), established at the National Institute of Health (Istituto Superiore di Sanità, ISS), is responsible for surveillance of the national program of iodoprophylaxis.

WHO also recommends limiting salt consumption to less than 5 g/day [5] to prevent arterial hypertension and the increased risk of stroke, coronary heart disease, and premature death [6]. Furthermore, the WHO/United Nations Global Non-Communicable Diseases (NCDs) 2013–2020 Action Plan indicated a 30% relative reduction in the mean population salt intake as one of the nine global targets to be reached by 2025 [7]. Then, salt consumption and iodine intake are clearly strictly interrelated dietary factors.

We have previously published the results of a survey carried out in 2008–2012 in a national sample of the Italian adult population, that showed an average salt consumption definitely higher than the standard dietary target of 5 g/day (or 2 g/day of sodium) [8,9] and concomitantly an inadequate iodine intake in both men and women and for all age categories [10].

According to WHO, an iodine concentration in the iodized salt between 20 and 40 mg/kg may allow an adequate iodine intake even at a salt intake of 5 g/day or below [3]. To test this hypothesis in real-life conditions, we conducted a comparative analysis of salt consumption and iodine intake in the sample of the Italian adult population mentioned above and estimated the relative contribution given by the consumption of both iodized salt and by the iodine introduced by foods to the total iodine intake.

## 2. Methods

### 2.1. Study Population and Survey Protocol

Within the CUORE Project, between 2008 and 2012, the Italian National Institute of Health (ISS) in collaboration with the National Association of Hospital Cardiologists (ANMCO), performed the Cardiovascular Epidemiology/Health Examination Survey (OEC/HES) [11]. Within the OEC/HES 2008–2012, sodium and iodine excretion were assessed in the framework of the Interdisciplinary Group for the Reduction of Salt Intake in Italy (MINISAL-GIRCSI Programme) and the Meno Sale Più Salute Programme. A detailed description of the methods for data collection and measurement of electrolyte excretion has been previously provided by Donfrancesco et al. [12] and by Iacone et al. [10]. For the purpose of the present study, a total of 9111 participants aged 25–79 years, 4555 men and 4556 women, from twenty-three centres of all the Italian regions were considered. The mean participation rate was 53%.

The study participants completed a questionnaire covering demographic information, performed a 24-h urine collection, underwent a standard physical examination and an anthropometric evaluation, including measurement of height and weight and calculation of Body Mass Index (BMI), and completed the EPIC food frequency questionnaire [13] for the assessment of their habitual food consumption. The survey was approved by the Ethical Committee of the ISS on 11 November 2009.

**Figure 1:**
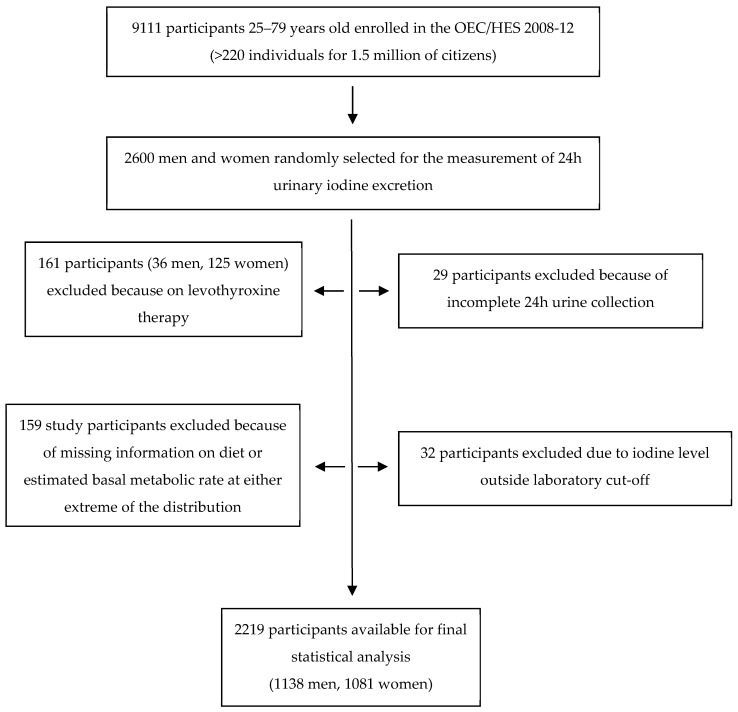
Flowchart of the study population.

Twenty-six hundred participants of both sexes and all the Italian regions (about 130 participants per region) were randomly selected from the entire cohort for the urinary iodine measurement. Twenty-nine subjects were then excluded from the analysis because of suspected incomplete 24-h collection (participant report of incomplete urine collection, 24 h urine volume below 500 mL, or creatinine content referred to body weight lower than the mean minus two standard deviations from the population mean [8]). Twenty-four subjects were excluded because of a urinary iodine concentration (UIC) below the analytical sensitivity of the method (<5 µg/L), eight subjects because their UIC was >400 µg/L, presumably due to occasional high iodine ingestion (food with very high iodine content, local antiseptic, disinfectants, or iodine-containing toothpaste). Another 161 participants (36 men and 125 women) were excluded because of ongoing chronic treatment with levothyroxine. Finally, 159 participants were excluded because of inadequate food questionnaire compilation: participants with missing information on diet (*n* = 127), with a ratio of total energy intake (determined from the questionnaire) to estimated basal metabolic rate at either extreme of the distribution (using first and last half-percentiles as cut-offs; 11 males and 12 females), or with more than 50% missing food items in the FFQ (five males and four females). Eventually, 2219 participants were included in the analysis (1138 men and 1081 women) (Figure 1).

### 2.2. Study Procedures

#### 2.2.1. Protocol for the Estimation of Salt Consumption and Iodine Intake

Each study participant received a plastic container for the 24-h urine collection, together with detailed instructions on how to collect complete 24-h urine. The participants performed a urine collection discarding the first void in the morning and then collecting all the urines until the following morning, including the first void. Once the collection was returned, the total volume of the urine was recorded, and three samples were extracted after shaking. The samples were immediately stored in plastic containers and frozen at −80 °C for later analyses. Compliance with the request to provide a 24 h urine collection was 92%.

Sodium and creatinine measurements were carried out centrally at the Department of Clinical Medicine and Surgery, Federico II University of Naples Medical School, as previously reported [8]. Urinary iodine was analysed in the laboratories of the Department of Translational Medical Science, Federico II University of Naples, by an Autoanalyzer 3 system (Bran + Luebbe GmbH, Norderstedt, Germany), using the ceric-arsenious acid reaction and digestion method by ultraviolet irradiation [14]. Urinary iodine excretion (UIE) was expressed as micrograms per 24 h. Daily iodine intake (DII) was estimated in µg/day by the formula: UIE/0.92, assuming that 92% of the ingested iodine is excreted in the urine [15,16]. The prevalence of inadequate iodine intake in the study population was assessed according to the European Food Safety Authority (EFSA) adequate intake (AI) value for iodine (150 µg/day for adults) [16]. Urinary sodium excretion was expressed as millimoles per 24 h. Daily salt consumption was estimated in grams/day by the formula: (Urinary sodium excretion/1000) × 58.44/0.90, where 58.44 is the NaCl molar mass, considering a recovery rate based on the broad assumption that 90% of the sodium ingested is excreted in the urines [17].

The prevalence of excess sodium (salt) consumption in the study population was assessed based on the maximum daily salt intake recommended by WHO [5].

Urinary creatinine, measured by the kinetic Jaffé reaction using an ABX Pentra 400 apparatus (HORIBA ABX, Rome, Italy), was used as an indicator to assess the adequacy of the 24-h collection, as mentioned above. Quality controls were performed using the Urichem Gold Standards from Bio Development SRL (Milan, Italy).

#### 2.2.2. Estimation of Dietary Iodine and Iodized Salt Consumption

The iodine content of the food items included in the EPIC FFQ [13] (Supplementary Table S1) was obtained by the Italian Food Composition Tables for Epidemiological Studies [18] or by the work of Pastorelli et al. who directly analysed the iodine content of most food samples representative of the Italian eating habits [19].

The estimation of the total iodine provision by food and by iodized salt was made considering a 30% iodine loss occurring through cooking and stocking [20]. The amount of iodine deriving from the use of salt was estimated by subtracting the iodine provided by food from the total iodine uptake. The amount of iodized salt (g/day) consumed by each study participant was calculated by dividing the iodine intake from salt by 21 (the micrograms of iodine provided by 1 g of iodized salt accounting for the 30% losses). The intake of iodized salt was also expressed as a percentage of the total salt intake.

### 2.3. Statistical Analysis

Statistical analysis was performed using the Statistical Package for the Social Sciences (SPSS Statistics for Windows, Version 27.0; IBM Corp, Armonk, NY, USA). The descriptive statistics covered the whole study population and the population stratified by gender and quintiles of salt intake. Results were expressed as median and interquartile range for the descriptive statistics since the health authorities reference values and most articles dealing with iodine status in other countries use medians. To estimate the amount and proportion of iodine provided by salt, total iodine intake and iodine coming from food were additionally reported as mean (Figure 3) since medians cannot be algebraically subtracted from one another. For categorical variables, the results were reported as frequencies (%). Non-parametric tests were used to test between-group differences (Mann-Whitney test in the case of two groups and Kruskal-Wallis test for more than two groups). Linear regression analysis between total salt and iodine intake was not performed in our population as the canonical assumptions of linear regression were not all met. The Jonckheere–Terpstra test was used for the analysis of the trend, and Spearman rank correlation analysis to evaluate the possible associations among the variables under investigation. Two-sided *p* values less than 0.05 were considered statistically significant.

## 3. Results

Table 1 shows median and interquartile range (IQR) for age, BMI, salt consumption, and iodine intake of male (*n* = 1138) and female participants (*n* = 1081), and of the whole study population (*n* = 2219).

Overall, there were 94.7% of study participants with a salt intake greater than 5 g/day and 71.5% of participants with an iodine intake below the EFSA AI for iodine (<150 µg/day). Female participants had a lower BMI, consumed a lower daily amount of salt, and had a lower daily intake of iodine as compared with male participants. Accordingly, participants with a salt intake exceeding 5 g/day were 91.5% among women and 97.6% among men (chi-square = 41.2, *p* < 0.001) whereas those with iodine intake below the AI were 78.7% among women and 64.8% among men (chi-square = 52.4, *p* < 0.001).

Salt consumption and iodine intake were directly associated (Spearman rho = 0.341; *p* < 0.001). Salt intake was not significantly related to age (Spearman rho = −0.042, *p* = 0.050), while it was directly associated to BMI (Spearman rho = 0.226, *p* < 0.001). Iodine intake was weakly and inversely associated with age (Spearman rho = −0.053, *p* = 0.013) and weakly and directly associated with BMI (Spearman rho = 0.092, *p* < 0.001). Partial correlation analysis indicated that the direct association between salt consumption and iodine intake was attenuated but remained statistically significant when accounting for sex, age, and BMI (Spearman rho = 0.298, *p* < 0.001).

Upon stratification of the study population by quintile of salt consumption, a progressive increase in iodine intake was apparent across quintiles with a significant trend (Jonckheere–Terpstra test for trend, *p* < 0.001) in the whole population and both male and female participants (Table 2). With reference to the EFSA AI value for iodine, median iodine intake was below the adequacy level in the whole population and male participants up to the fourth quintile of salt consumption and in female participants in all quintiles.

**Figure 2:**
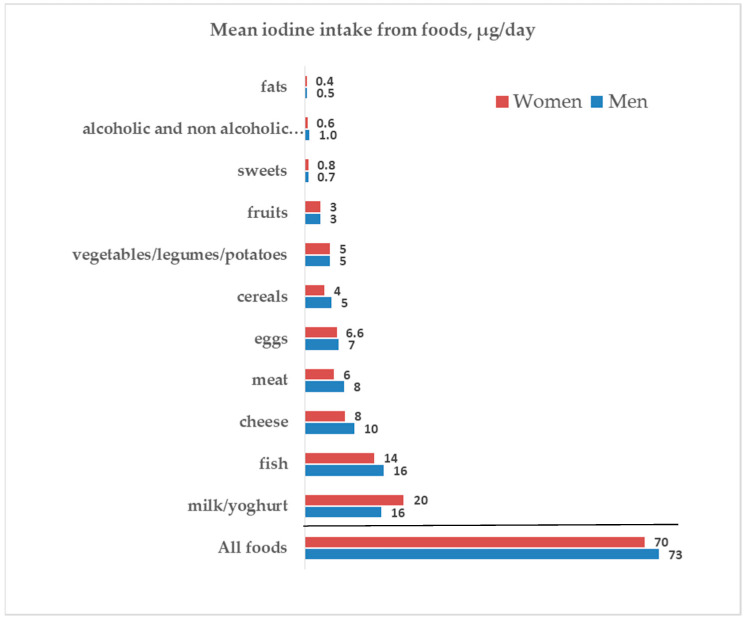
Iodine intake from foods and by sex.

Iodine intake derives both from food and from iodized salt. We estimated the respective amounts as described in Section 2. Iodine contributions from different food groups and by sex are shown in Figure 2 and Supplementary Table S1. Milk/yoghurt, fish, cheese, meat, and eggs were the major suppliers of iodine from food in both male and female participants. The iodine provision by food referred to energy intake was 49 (39–57) µg/1000 Kcal in men and 56 (44–65) µg/1000 Kcal in women (*p* < 0.001).

**Figure 3:**
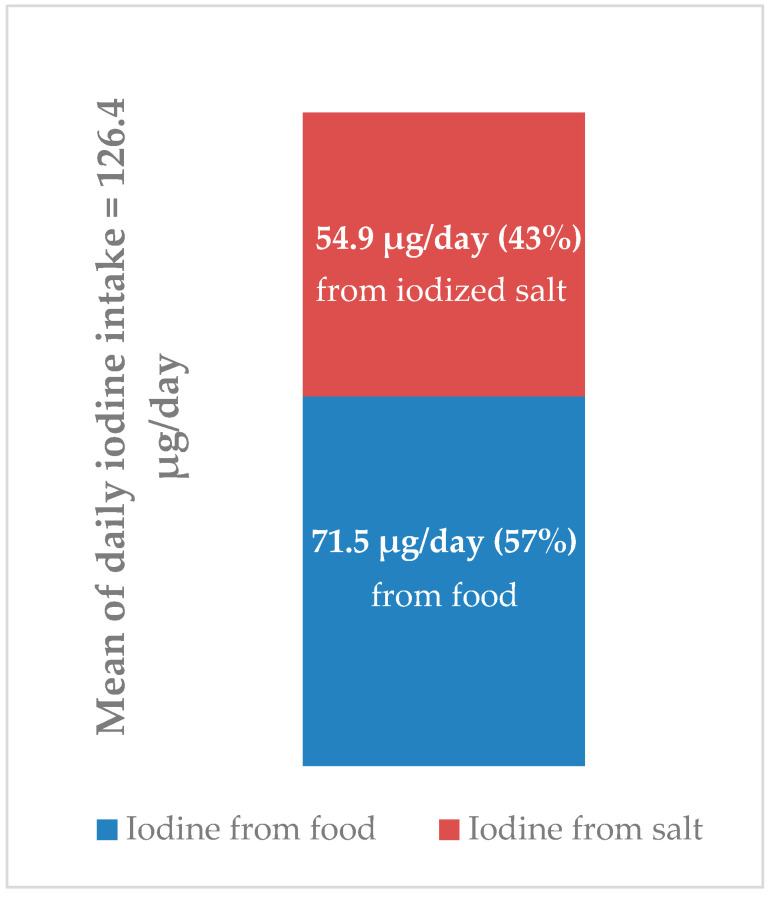
Estimates of iodine intake from food and iodized salt and their respective proportions.

As explained in Section 2, the percentages of iodine deriving respectively from food and iodized salt in the whole population were estimated based on the data of total iodine intake (126.4 µg/day) and the amount of iodine provided by food (71.5 µg/day), both expressed as mean since the medians cannot be algebraically subtracted from one another. Total iodine intake was assessed from 24 h urinary excretion, whereas iodine intake from food was estimated from the EPIC FFQ. An estimate of the amount of iodine provided by iodized salt (54.9 µg/day) was obtained by the difference between the total iodine intake and iodine intake from food (Figure 3).

**Figure 4:**
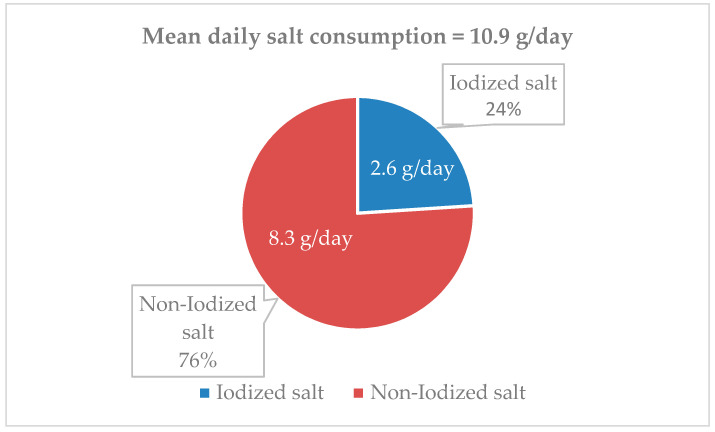
Proportion of estimated iodized salt intake referred to the total salt consumption.

As described in Section 2 (“Estimation of dietary iodine and iodized salt consumption”), the average amount of iodized salt consumed by the whole population (2.6 g/day) was estimated by dividing the iodine intake from salt (54.9 µg/day) by 21 (the micrograms of iodine provided by 1 g of iodized salt accounting for 30% losses). The intakes of iodized and non-iodized salt were also depicted as a percentage of the daily total salt intake (Figure 4).

## 4. Discussion

A reduction of daily salt consumption and an appropriate intake of iodine through the iodization of food-grade salt are two milestones of the WHO population strategy aimed at the prevention of cardiovascular diseases and respectively of thyroid disorders secondary to iodine deficiency states. According to WHO, these two policies are not conflictual with each other [3]. We have previously reported data on salt consumption [8] and iodine intake [10] in a national sample of Italian adult population. Purpose of the present analysis was to elucidate the relationship between salt consumption and iodine intake in the same population sample and to estimate the respective contributions of iodine intake from food and from iodized salt consumption to the achievement of a condition of adequate iodine status. The main results of our study are the following:

(1) iodine intake increased gradually with increasing salt intake. This trend was at least in part independent of sex, age and body weight;

(2) the estimated consumption of iodized salt was low, corresponding to 24% of the total salt intake, which includes both discretionary and non-discretionary salt;

(3) greater iodine intake and a lower probability of iodine inadequacy were achieved in the whole population and by men in the highest quintile of salt intake, at a median salt intake of as much as 16.7 and 18.1 g/day, respectively;

(4) the majority of those in the study population who complied with the WHO recommendation of a total salt intake of less than 5 g/day did not achieve an adequate iodine intake;

(5) men had a lower rate of iodine inadequacy than women, very likely as a consequence of their greater salt consumption;

(6) food consumption contributed for about 57% to the total iodine intake in the whole population, so iodized salt contributed for about 43%;

(7) milk and yogurt provided 22–29% while fish consumption 20–22% of the total iodine intake from food.

These results are in line with those recently reported for a national Italian paediatric population sample which was examined in the same period of the present survey. Both among children and adolescents [20] and male and female adults here reported the proportion of iodized salt out of total salt intake varied from a minimum of 16% to a maximum of 29% in different sex and age categories. Similar to the present study, also in the children and adolescent population an adequate iodine intake was reached only at the cost of a very high sodium intake with respect to WHO recommendation [20]. In the paediatric population, it was estimated that 72% of iodine was provided by food in children and in female adolescents, with a slightly lower figure (65%) in male adolescents [20]. In the present study, the provision of iodine from food for the whole population amounted to about 57%.

Our findings on the absolute amount of iodine provided by food in the adult population were only slightly higher than the estimates by Pastorelli et al. [19], taking account of the 30% iodine loss secondary to stocking and cooking of foods. The estimates by Pastorelli and coworkers were obtained with a different method, i.e., by directly measuring the iodine content of foods representing major sources of dietary iodine and matching the results with the median values of consumption of the same foods in the Italian adult population based on the 2005-6 INRAN-SCAI survey [21]. Based on their findings, Pastorelli and coworkers [19] hypothesized that an average intake of 5 g of iodized salt per day should be sufficient to meet the iodine adequacy level set by EFSA at 150 µg/day. This expectation would be reasonable if all the salt consumed by individuals, as either discretionary (salt added while cooking and eating) or non-discretionary salt (the one present in foods bought in shops and supermarkets or eaten out-of-home), was iodized. The results of the present survey make it clear that this was not the case in real life as iodized salt represented only 24% of the total salt intake. This amount was very likely almost entirely given by discretionary salt as the presence of iodized salt in commercially available processed foods in Italy is very low based on available information [22].

Based on the total salt consumption results and on the estimate by Leclercq and coworkers of a 35% discretionary salt intake in Italy [23], we estimate that discretionary salt consumption in our study population was approximately 4.3 g/day for men and 3.3 g/day for women, which is quite high with respect to the WHO recommendation of a 5-g total daily salt intake. It must be noticed that the results of an online survey conducted by the Italian Society of Human Nutrition (SINU) Group on an opportunistic sample of Italian general population indicated that the proportion of people who declare the regular use of iodized salt in the preparation and consumption of food at home (“discretionary” salt) may indeed not exceed 50% [24]. Approximately in the same period (2016–2019), another survey within the PASSI surveillance system of the National Institute of Health on 130,000 subjects showed that 41% of the people interviewed declare to use iodized salt “always”, 12% “often” and 18% sometimes [25].

This notwithstanding, even in the hypothesis that the amount of discretionary salt consumed in Italy was entirely represented by iodized salt, while adding the iodine provided by foods, it would barely allow to meet the iodine adequate intake (150 µg/day) in men but not in women. In conclusion, it appears that a sufficient supply of iodized salt could only be obtained by substantially increasing the use of iodized salt in commercially available processed foods as well as in the public and private restoration (non-discretionary salt).

In addition to the use of iodized salt, it is important to not disregard the other important sources of iodine given by food. According to the estimates by Pastorelli et al. [19], the food groups which mainly contribute to the iodine intake were, in decreasing order, fish, dairy products and cereals. In our survey, based on the 2008–2012 data provided by the administration of the EPIC food frequency questionnaire, milk and yoghurt were the main dietary sources of iodine, followed by fish and by cheese. Indeed, when comparing the reported food consumptions with the Italian Guidelines for a healthy nutrition [26], it appears that our study population was compliant with the recommendations about milk, yogurt, and cereals, whereas the consumption of fish, eggs, fruits, and vegetables was lower than that recommended. Indeed, with the hypothetical consumption of the suggested dietary amounts (fish > 2.5 portions/week; 2–4 eggs/week; 5 portions/day of fruits and vegetables) the iodine intake from food could rise by a further 10%.

It is worthy to compare the Italian data with those from other countries. Switzerland has a long history in iodoprophylaxis, being a pioneering country in the legal iodization of table salt, which was introduced as early as in 1922 [27]. Haldiman et al. [28], in a survey conducted between 2010 and 2012, found a median value of iodine intake of 148.3 µg/day, close to the EFSA AI, and estimated a contribution of iodised salt to total iodine intake of 54%: considering that the iodine fortification level of salt was 20 mg/kg, these data seem to indicate that the consumption of iodized salt was much higher than in Italy. In Germany, the iodization of food grade salt began in 1981 but only after a change in the legislation in 1993 facilitating the usage of iodized salt in processed foods, the iodine status significantly improved in the German paediatric population [29]. Heshe et al. [30] carried out a survey on a national representative sample of 6738 adults between 2008 and 2011 and reported a salt contribution of 42% to the total iodine intake, similar to our study.

Charlton et al. [31] investigated to what extent the South Africa’s mandatory salt reduction policy affected salt iodization programs. In a random sample of adult population, the authors found that, similarly to our findings, iodine excretion increased with increasing salt consumption and participants with salt consumption within the WHO limit of <5 g/day did not meet the estimated average requirement for iodine intake. Direct information on the possible effect of salt intake reduction on iodine intake was provided by a randomized controlled trial conducted in a Chinese children population [32] using 24 h urine collections: a 25% reduction in salt intake over three and half months was associated with a significant 19% reduction in iodine intake and with a decrease in the proportion of children meeting the estimated average requirement for iodine.

### Strengths and Limitations of the Study

Our work has several strengths as well as some limitations. It is to our knowledge the first report on the habitual sodium and iodine intake association assessed on a large national sample of the adult general population using gold standard methodologies. The measurement of sodium and iodine intake was based on 24-h urine collection, the recognized best proxy for this type of measurements. Many efforts were spent to ensure proper urine collection by the study participants, both providing them with detailed written instructions and later checking for the urine creatinine content in order to exclude from the analysis the individuals with creatinine excretion levels exceedingly low in relation to body weight. Participants on chronic therapy with levothyroxine were excluded as were those with unusually high levels of iodine excretion suggesting the occasional consumption of other iodine-rich drugs or foods or iodine supplements. In addition to the measurement of total iodine intake, we also directly estimated the amount of iodine provided by food using a validated food frequency questionnaire widely applied in epidemiological research. By so doing, we could indirectly also estimate the amount of iodine deriving from the use of iodized salt.

The major limitation is that our study population was recruited almost 10 years ago so our results depict the situation at that time: nevertheless, the current Italian legislation had been already in force for approximately 5 years at the time of the survey so its effects should have been sufficiently displayed. In any case, the present data are an important reference for the assessment of future changes in the contribution provided by iodized salt consumption as well as by food intake to the iodine status in Italy.

Another limitation of our study was the use of a single urine collection to estimate the participants’ habitual salt and iodine intake: it is known that repeated 24 h collections are needed to estimate the “habitual” iodine intake with sufficient accuracy at the individual level [33]. For this reason, we refer in our report only to the median or mean salt and iodine intakes of the population as a whole or of population subgroups without attempting to describe the iodine intake or iodine status of the individual participants.

## 5. Conclusions and Perspectives

In summary, we reported that in this national sample of adult general population examined between 2008 and 2012 a significant relationship occurred between sodium and iodine intake and that an adequate iodine intake depended on the consumption of large amounts of salt: in order of importance, this probably was a consequence of the negligible amount of iodine provided by commercially available salt-rich processed foods, by the insufficient use of iodized salt at home and by the less-than-optimal consumption of iodine rich foods.

It is an element of concern that in our study population compliance with the WHO recommendations about salt intake was associated with a large proportion of iodine inadequacy. This is all the more important as gradual reduction of salt intake at the population level is being currently observed in several countries, including Italy [12], thus increasing the potential for insufficient intake of iodized salt. We think that these problems may be common to other countries with iodoprophylaxis policies similar to the one adopted in Italy. In keeping with the results of recent studies carried out in other countries [28,29,30], our data point to the need to continue to invest in vigorous policies encouraging the use of less salt but only iodized salt not only by households but also by the catering system and, even more important, by the food production system being the salt content of processed foods the major source of salt in the current human diet. Indeed, despite the efforts of the public health institutions and the iodoprophylaxis campaigns, the sale of non-iodized salt is still unrestricted in all food markets and, most importantly, the use of iodized salt in food manufacturing at both artisanal and industrial level remains marginal based on available information (22). Additional strategies to prevent iodine deficiency could be an increase in the iodine fortification of animal food and the promotion of an increased consumption of iodine-rich foods, in particular milk, dairy products, and fish: the latter would be consistent with the implementation of a healthy diet for the prevention of non-communicable diseases.

## Figures and Tables

**Table 1:** Medians (IQR) of age, BMI, salt, and iodine intake in the overall study population and by sex.

	Whole Population *n* = 2219	Men *n* = 1138	Women *n* = 1081	*p* *
Age, years	56 (46–66)	56 (45–66)	57 (46–67)	0.304
BMI, kg/m^2^	26.7 (24.0–30.2)	27.1 (24.9–30.2)	26.1 (23.0–30.2)	<0.001
Salt consumption, g/day	10.2 (7.8–13.3)	11.7 (8.9–14.9)	9.1 (6.8–11.5)	<0.001
Iodine intake, µg/day	95 (51–165)	110 (60–189)	84 (43–138)	<0.001

* Mann-Whitney U-test for independent samples (male vs. female participants).

**Table 2:** Medians (IQRs) of salt (g/day) and iodine intake (µg/day) by quintiles of salt consumption and by sex.

Whole Population	I Quintile	II Quintile	III Quintile	IV Quintile	V Quintile
	*n* = 444	*n* = 444	*n* = 444	*n* = 443	*n* = 444
Salt intake	5.8 (4.9–6.5)	8.3 (7.8–8.8)	10.2 (9.7–10.8)	12.6 (11.9–13.3)	16.7 (15.3–19.3)
Iodine intake	61 * (29–105)	82 * (48–130)	96 * (51–154)	111 * (67–177)	148 (78–231)
Men	I quintile	II quintile	III quintile	IV quintile	V quintile
	*n* = 228	*n* = 228	*n* = 227	*n* = 227	*n* = 228
Salt intake	6.8 (5.7–7.4)	9.4 (8.9–9.9)	11.7 (11.2–12.4)	14.0 (13.5–14.9)	18.1 (16.6–21.1)
Iodine intake	68 * (37–113)	93 * (55–152)	119 * (71–179)	134 * (75–201)	174 (85–240)
Women	I quintile	II quintile	III quintile	IV quintile	V quintile
	*n* = 216	*n* = 216	*n* = 217	*n* = 216	*n* = 216
Salt intake	5.3 (4.2–5.8)	7.3 (6.8–7.8)	9.1 (8.6–9.5)	10.9 (10.3–11.5)	14.2 (12.9–16.4)
Iodine intake	52 * (25–95)	78 * (44–120)	91 * (52–147)	89 * (45–148)	120 * (66–186)

* Values significantly below the EFSA adequate iodine intake (150 µg/day), *p* < 0.001 (one sample Wilcoxon signed rank test).

## Data Availability

Not applicable.

## Supplementary Materials

The following are available online at https://www.mdpi.com/article/10.3390/nu13103486/s1, Table S1: Iodine content of food groups.

## References

[1] World Health Organization (WHO) (2014). WHO Guideline: Fortification of Food-Grade Salt with Iodine for the Prevention and Control of Iodine Deficiency Disorders.

[2] WHO, UNICEF, ICCIDD (2007). Assessment of Iodine Deficiency Disorders and Monitoring Their Elimination.

[3] (2007). Salt as a Vehicle for Fortifcation. Report of a WHO Expert Consultant.

[4] Law 21 March 55/2005. https://www.gazzettaufficiale.it/eli/gu/2005/04/20/91/sg/pdf.

[5] WHO Reducing Salt Intake in Populations: Report of a WHO Forum and Technical Meeting World Health Organization 2007. https://www.who.int/dietphysicalactivity/Salt_Report_VC_april07.pdf.

[6] D’Elia L., Galletti F., La Fata E., Sabino P., Strazzullo P. (2018). Effect of dietary sodium restriction on arterial stiffness: Systematic review and meta-analysis of the randomized controlled trials. J. Hypertens..

[7] WHO (2013). Global Action Plan for the Prevention and Control of Noncommunicable Diseases 2013–2020.

[8] Donfrancesco C., Ippolito R., Lo Noce C., Palmieri L., Iacone R., Russo O., Vanuzzo D., Galletti F., Galeone D., Giampaoli S. (2013). Excess dietary sodium and inadequate potassium intake in Italy: Results of the MINISAL study. Nutr. Metab. Cardiovasc. Dis..

[9] Cappuccio F.P., Ji C., Donfrancesco C., Palmieri L., Ippolito R., Vanuzzo D., Giampaoli S., Strazzullo P. (2015). Geographic and socioeconomic variation of sodium and potassium intake in Italy: Results from the MINISAL-GIRCSI programme. BMJ Open.

[10] Iacone R., Iaccarino Idelson P., Formisano P., Russo O., Lo Noce C., Donfrancesco C., Macchia P.E., Palmieri L., Galeone D., di Lenarda A. (2021). Iodine Intake Estimated by 24h Urine Collection in the Italian Adult Population: 2008–2012 Survey. Nutrients.

[11] Giampaoli S., Palmieri L., Donfrancesco C., Lo Noce C., Pilotto L., Vanuzzo D. (2015). Osservatorio Epidemiologico Cardiovascolare/Health Examination Survey Research Group. Cardiovascular health in Italy. Ten-year surveillance of cardiovascular diseases and risk factors: Osservatorio Epidemiologico Cardiovascolare/Health Examination Survey 1998–2012. Eur. J. Prev. Cardiol..

[12] Donfrancesco C., Lo Noce C., Russo O., Minutoli D., Di Lonardo A., Profumo E., Buttari B., Iacone R., Vespasiano F., Vannucchi S. (2020). Trend of salt intake measured by 24-h urine collection in the Italian adult population between the 2008 and 2018 CUORE Project surveys. NMCD.

[13] Pisani P., Faggiano F., Krogh V., Palli D., Vineis P., Berrino F. (1997). Relative validity and reproducibility of a food frequency dietary questionnaire for use in the Italian EPIC centres. Int. J. Epidemiol..

[14] Campanozzi A., Rutigliano I., Macchia P.E., De Filippo G., Barbato A., Iacone R., Russo O., D’Angelo G., Frigeri M., Pensabene L. (2019). Iodine deficiency among Italian children and adolescents assessed through 24-hour urinary iodine excretion. Am. J. Clin. Nutr..

[15] Institute of Medicine (2001). Dietary Reference Intakes for Vitamin A, Vitamin K, Arsenic, Boron, Chromium, Copper, Iodine, Iron, Manganese, Molybdenum, Nickel, Silicon, Vanadium, and Zinc.

[16] EFSA NDA Panel (EFSA Panel on Panel on Dietetic Products Nutrition and Allergies) (2014). Scientific Opinion on Dietary Ref-erence Values for iodine. EFSA J..

[17] Cogswell M.E., Maalouf J., Elliott P., Loria C.M., Patel S., Bowman B.A. (2015). Use of Urine Biomarkers to Assess Sodium Intake: Challenges and Opportunities. Annu. Rev. Nutr..

[18] Salvini S., Parpinel M., Gnagnarella P., Maissoneuve P., Turrini A. (1998). Banca Dati di Composizione Degli Alimenti Per Studi Epidemiologici in Italia.

[19] Pastorelli A.A., Stacchini P., Olivieri A. (2015). Daily iodine intake and the impact of salt reduction on iodine prophylaxis in the Italian population. Eur. J. Clin. Nutr..

[20] Iacone R., Iaccarino Idelson P., Campanozzi A., Rutigliano I., Russo O., Formisano P., Galeone D., Macchia P.E., Strazzullo P. (2020). MINISAL-GIRCSI Study Group. Relationship between salt consumption and iodine intake in a pediatric population. Eur. J. Nutr..

[21] Leclercq C., Arcella D., Piccinelli R., Sette S., Le Donne C., Turrini A. (2009). INRAN-SCAI 2005-06 Study Group. The Italian National Food Consumption Survey INRAN-SCAI 2005-06: Main results in terms of food consumption. Public Health Nutr..

[22] Olivieri A., Vitti P. (2014). Istituto Superiore Di Sanità. Rapporti ISTISAN 14/6. Attività Di Monitoraggio Del Programma Nazionale per la Prevenzione Dei Disordini Da Carenza Iodica. Epidemiologia E Sanità Pubblica. http://www.salute.gov.it/imgs/C_17_pubblicazioni_2375_allegato.pdf.

[23] Leclercq C., Ferro-Luzzi A. (1991). Total and domestic consumption of salt and their determinants in three regions of Italy. Eur. J. Clin. Nutr..

[24] Iaccarino Idelson P., D’Elia L., Cairella G., Sabino P., Scalfi L., Fabbri A., Galletti F., Garbagnati F., Lionetti L., Paolella G. (2020). On Behalf Of The Sinu-Gircsi Working Group. Salt and Health: Survey on Knowledge and Salt Intake Related Behaviour in Italy. Nutrients.

[25] Istituto Superiore di Sanità L’epidemiologia per La Sanità Pubblica: Sorveglianza PASSI. https://www.epicentro.iss.it/passi/dati/sale.

[26] Consiglio per La Ricerca in Agricoltura E L’analisi Dell’economia Agraria (CREA) (2018). Linee Guida per Una Sana Alimentazione. Revisione. https://www.crea.gov.it/documents/59764/0/LINEE-GUIDA+DEFINITIVO.pdf/28670db4-154c-0ecc-d187-1ee9db3b1c65?t=1576850671654.

[27] Zimmermann M.B., Aeberli I., Torresani T., Bürgi H. (2005). Increasing the iodine concentration in the Swiss iodized salt program markedly improved iodine status in pregnant women and children: A 5-y prospective national study. Am. J. Clin. Nutr..

[28] Haldimann M., Bochud M., Burnier M., Paccaud F., Dudler V. (2015). Prevalence of iodine inadequacy in Switzerland assessed by the estimated average requirement cut-point method in relation to the impact of iodized salt. Public Health Nutr..

[29] Remer T., Neubert A. (1998). A never-ending story of an insufficient iodine status without mandatory iodization of foods? A German experience. J. Clin. Endocrinol. Metab..

[30] Esche J., Thamm M., Remer T. (2020). Contribution of iodized salt to total iodine and total salt intake in Germany. Eur. J. Nutr..

[31] Charlton K., Ware L.J., Menyanu E., Biritwum R.B., Naidoo N., Pieterse C., Madurai S., Baumgartner J., Asare G.A., Thiele E. (2016). Leveraging ongoing research to evaluate the health impacts of South Africa’s salt reduction strategy: A prospective nested cohort within the WHO-SAGE multicountry, longitudinal study. BMJ Open.

[32] He F.J., Wu Y., Feng X.X., Ma J., Ma Y., Wang H., Zhang J., Yuan J., Lin C.P., Nowson C. (2015). School based education programme to reduce salt intake in children and their families (School-Edu Salt): Cluster randomised controlled trial. BMJ.

[33] König F., Andersson M., Hotz K., Aeberli I., Zimmermann M.B. (2011). Ten repeat collections for urinary iodine from spot samples or 24-hour samples are needed to reliably estimate individual iodine status in women. J. Nutr..

