# Supplementary material for: Iodine Intake from Food and Iodized Salt as Related to Dietary Salt Consumption in the Italian Adult General Population
Source: Nutrients. 2021 Sep 30;13(10):3486. doi: 10.3390/nu13103486 (PMC8537510; doi:10.3390/nu13103486)
Supplement: Supplementary file 1 [file nutrients-13-03486-s001.zip › nutrients-1393200-supplementary.pdf]

Supplementary Tables S1. Iodine content of food groups.

Iodine content of food groups derived from the IEO food composition tables (ref. 18) or from the analysis of food samples by Pastorelli et al. (ref. 19, signalled by the asterisc), mean food consumption assessed by the EPIC food frequency questionnaire (ref. 13), and iodine intake by food group. Note that 30% of iodine is lost by cooking and inappropriate storage.

The mean iodine content and the mean consumption for the food groups that include more than one type of food (as fish + crustaceans + molluscs) are the weighted average of the iodine content and the food consumption of the respective food group components.

MEN	Iodine content (µg/100g)	Mean food consumption (g/day)	Mean Iodine intake (µg/day)	Mean Iodine intake – 30% (µg/day)	Percent contribution to iodine intake
**Fish/crustaceans and molluscs**	62.0	41.3	23.1	16.2	22.1
**Milk/Yogurt***	15.0	149.6	22.4	15.7	21.5
**Cheese (fresh and matured)**	27.4	58.8	14.6	10.2	14.0
**Meat (beef, veal, chicken etc.)**	9.1	141.2	11.5	8.1	11.0
**Eggs**	53.0	18.7	9.9	6.9	9.5
**Cereals (pasta, bread and other grains)***	3.2	236.4	7.8	5.4	7.4
**Vegetables/ Legumes/potatoes***	3.0	245.1	7.4	5.2	7.1
**Fruits**	2.5	278.3	4.7	3.3	4.4
**Sweets**	1.7	41.9	1.0	0.7	1.0
**Alcoholic/Non alcoholic beverages**	0.3	476.1	1.4	1.0	1.4
**Fats**	8.0	32.4	0.7	0.5	0.6
**Total**			**104.5**	**73.2**	**100**

WOMEN	Iodine content (µg/100g)	Mean food consumption (g/d)	Mean Iodine intake (µg/d)	Mean Iodine intake – 30% (µg/d)	Percent contribution to iodine intake
**Milk/Yogurt**	15.0	194.1	29.1	20.4	29.2
**Fish/crustaceans and molluscs**	62.0	36.6	20.4	14.3	20.4
**Cheese (fresh and matured)**	27.4	50.0	11.8	8.2	11.8
**Eggs**	53.0	17.7	9.4	6.6	9.4
**Meat (beef, veal, chicken etc.)**	9.1	108.1	8.6	6.1	8.7
**Vegetables/ Legumes/potatoes**	3.0	245.4	7.4	5.2	7.4
**Cereals (pasta, bread and other grains)**	3.2	194.4	5.8	4.1	5.8
**Fruits**	2.5	296.8	4.7	3.3	4.7
**Sweets**	1.7	40.1	1.1	0.8	1.1
**Alcoholic/Non alcoholic beverages**	0.3	298.5	0.9	0.6	0.9
**Fats**	8.1	29.9	0.6	0.4	0.6
**Total**			**99.8**	**70.0**	**100**
